# Supplementary material for: Four new species of dragon pseudoscorpions (Pseudoscorpiones: Pseudotyrannochthoniidae: Spelaeochthonius) from caves in South Korea revealed by integrative taxonomy
Source: PLoS One. 2025 Jul 9;20(7):e0325375. doi: 10.1371/journal.pone.0325375 (PMC12240370; doi:10.1371/journal.pone.0325375)
Supplement: Attachment — Submitted filename: Spelaeochthonius KH5.docx [file pone.0325375.s001.docx]

**Four new species of dragon pseudoscorpions (Pseudoscorpiones: Pseudotyrannochthoniidae: *Spelaeochthonius*) from caves in South Korea revealed by integrative taxonomy**

Kyung–Hoon Jeong^1,2^, Danilo Harms^3,4,5^, Jung–sun Yoo^6^, and Sora Kim^1,2^

^1^Department of Agricultural Convergence Technology, Jeonbuk National University, Jeonju, Republic of Korea

^2^Lab of Insect Phylogenetic & Evolution, Jeonbuk National University, Jeonju, Republic of Korea

^3^Museum of Nature Hamburg – Zoology, Institute for the Analysis of Biodiversity Change, Hamburg, Germany

^4^Research Associate, Harry Butler Institute, Murdoch University, Murdoch, Australia

^5^Research Fellow, Department of Zoology and Entomology, University of the Free State, Bloemfontein 9301, South Africa.

^6^Wild Animal Quarantine Center, Incheon, Republic of Korea

Corresponding author: skim01@jbnu.ac.kr

**1. Abstract**

Karst research in Korea is still in its infancy and the invertebrate fauna of subterranean systems across the country is poorly known. One of the very diverse lineages in caves across Korea, the pseudoscorpions, are almost undocumented although they represent stunning examples of cave adaptations and troglomorphism. In this study, we provide a phylogenetic hypothesis for the pseudoscorpion *Spelaeochthonius* Morikawa, 1954 (Pseudoscorpiones: Pseudotyrannochthoniidae) in South Korea; a genus that exclusively occurs in caves across China, Japan and the Korean Peninsula. We report seven species of which four are newly described and illustrated based on molecular, distributional and morphological data: *Spelaeochthonius dugigulensis* **sp. nov.**, *S. geumgulensis* **sp. nov.**, *S. magwihalmigulensis* **sp. nov**. and *S. yamigulensis* **sp. nov**. All species are strongly cave-adapted and known from a single cave or karst system only, emphasizing the need to implement conservation strategies for Korean karst systems and their fauna.

Keywords: caves, DNA barcoding, false scorpions, Korean Peninsula, systematics, taxonomy.

**2. Introduction**

Recent studies have shown that South Korea may have more than 1,000 caves of which a significant proportion remains unexplored (Woo & Kim 2018). These caves encompass limestone cave systems and lava tubes spread across two extensive mountain ranges, the Taebaek Mountain Range and the Sobaek Mountain Range. These mountain ranges have served as refugia during glacial periods and as barriers to speciation in many animal lineages (Min et al. 2005; Lee et al. 2008; Kim et al. 2013; Kim et al. 2017) but little is still known about subterranean fauna that occurs beneath the surface in caves of different geological age and origin (Lee & Min 2021; You et al. 2022; Chang et al. 2022).

One of the most diverse animal groups in karstic caves across the Korean Peninsula is pseudoscorpions (Arachnida: Pseudoscorpions) but this fauna has not been studied until recently (You et al*.* 2022). In Korea, pseudoscorpions are a "neglected" invertebrate taxon with presently 28 species in 13 genera that belong to eight families (World Pseudoscorpiones Catalog 2024). However, a recent preliminary barcoding study has indicated that the species biodiversity in surface habitats must be significantly higher (Ohira et al. 2018). The subterranean fauna is probably also diverse, but to date, only five pseudoscorpion species have been recorded from caves in South Korea (Morikawa 1970; You et al. 2022). This is in sharp contrast to other countries in the region such as China where many new pseudoscorpion species are described each year, many of which were sampled from caves (Feng et al. 2020; Hou et al. 2022; Gao et al. 2023).

To date, all troglomorphic (strongly cave-adapted) pseudoscorpions in Korea belong to a single genus and family (Pseudotyrannochthoniidae: *Spelaeochthonius* Morikawa, 1954) that occurs in Korea with three described species: *Spelaeochthonius cheonsooi* You, Yoo, Harvey, and Harms, 2022; *S. dentifer* (Morikawa, 1970); and *S. seungsookae* You, Yoo, Harvey, and Harms, 2022 (Fig. 1). This genus is generally interesting because it is widespread in eastern Asia and has eleven species in China, Japan and Korea. All species are strongly cave-adapted and troglomorphic, meaning that they lack eyes and body pigmentation. The genus is replaced in surface habitats by the related genus *Allochthonius* Chamberlin, 1929 in the same family, and *Spelaeochthonius* might be an ancient relict lineage that was more widespread in the past but is now confined to subterranean refugia. Recent field work in caves across the Korean Peninsula suggests that *Spelaeochthonius* is indeed very diverse in South Korea and shows extreme degrees of regional endemism, but there are also practical problems because species are often rare in their natural habitats and difficult to sample (You et al*.* 2022).

This paper is part of an ongoing systematic study of pseudoscorpions in Korea (Jeong et al. 2024a, b) and adds to our knowledge of *Spelaeochthonius* on the Korean Peninsula by describing four new species from South Korea based on morphological and molecular analyses. All species are single-karst endemics and therefore of conservation relevance. We also provide a preliminary phylogeny for *Spelaeochthonius* using molecular sequences of the Cytochrome Oxidase subunit I (COI) “barcoding” gene, which is commonly used for species delimitation in pseudoscorpions (Muster et al. 2021; Ohira et al. 2018; Hlebec et al. 2023, 2024), and an updated distribution map for the genus in eastern Asia.

**3. Materials and methods**

**3.1. Field sampling and morphological identification**

All specimens used for taxonomy were collected from four caves located in the Gangwon-Province (GW), Gyeongsangbuk-Province (GB), and Chungcheongbuk-Province (CB) and preserved directly in 70% ethanol. Identification was conducted using a Leica Z16 APO stereomicroscope, with images captured using a Leica Z16 APO stereomicroscope and Dhyana 400DC (4M) sCMOS camera (TUCSEN, Fuzhou, China) using Mosaic Analysis Software 2.4. Images were edited using Adobe Photoshop 2024 (Adobe Inc.) and illustrations were drawn using Adobe Illustrator 2024 (Adobe Inc.). The format for the description follows previous papers (Jeong et al. 2024a, b) and distribution maps were generated using QGIS desktop 3.34.0 (OSGeo). Measurements and terminology follow Chamberlin (1931), Harvey (1991), Judson (2007) and Kolesnikov et al. (2023). Abbreviations for chelal trichobothria: ***b*** – basal, ***sb*** – subbasal, ***st*** – subterminal, ***t*** – terminal, ***ib*** – internal basal, ***isb*** – internal subbasal, ***eb*** – external basal, ***esb*** – external subbasal, ***it*** – internal terminal, ***ist*** – internal subterminal, ***et*** – external terminal, ***est*** – external subterminal. Abbreviations for carapacal setae: ***Ao*** – anterior ocular seta, ***Al*** – anterolateral seta, ***Am*** – anteromedial seta, ***Ol*** – lateral ocular seta, ***Om*** – medial ocular seta, ***Ml*** – medial ocular seta, ***Il*** – intermedialateral seta, ***Pl*** – posterolateral seta, ***Pm*** – posteromedial seta. To safeguard biologically sensitive cave habitats, the coordinates of the caves have been rounded to the nearest minute. This measure ensures that the precise locations remain protected from potential disturbances.

**3.2. Molecular methods and analyses**

A total of 14 sequences were used for molecular phylogenetic analyses of the mitochondrial Cytochrome Oxidase subunit I (mtCOI) “barcoding” gene, of which nine were newly generated in this study. Published sequences for *S. cheonsooi*, *S. dentifer*, *S. seungsookae*, *S. kobayashii*, and *Spelaeochthonius* sp. were sourced from NCBI (GenBank accession no. MZ394005–MZ394007, OR290003, OR290018), and eight new sequences were identified, corresponding to *S. dugigulensis* **sp. nov.**, *S. geumgulensis* **sp. nov.**, *S. magwihalmigulensis* **sp. nov.**, and *S. yamigulensis* **sp. nov** (Genbank accession no. xxxxxxx – to be added after manuscript acceptance). An additional sequence of *Allochthonius buanensis* Lee, 1982 was used as an outgroup.

DNA extraction was performed using two to three legs detached from each specimen with the DNeasy Blood & Tissue Kit (QIAGEN, Valencia, CA) and LaboPass^Tm^ DNA Purification Kit (Cosmo Genetech Co. Ltd., Seoul, South Korea) following the manufacturer’s protocol. PCR amplification for a 650 bp fragment of the COI gene was achieved by using the primer set LCO 1490 (5’-GGTCAACAAATCATCATAAAGATATTGG-3’) (Folmer et al. 1994) and HCOoutout (5’-GTAAATATATGRTGDGCTC-3’) (Prendini et al. 2005) and under the following conditions: initial denaturation at 94˚C for 2 min, followed by 40 cycles of denaturation at 95˚C for 30 sec, annealing at 40–45˚C for 30 sec, extension at 72˚C for 1 min, and a final extension at 72˚C for 5 min. DNA amplification utilized AccuPower PCR Premix (Bioneer, Daejeon, South Korea) and PCR products were examined in 1.2% agarose gels and sequenced at Macrogen, Inc. (Geumcheon–Gu, Seoul, South Korea).

Intra– and interspecific pairwise genetic distance calculations were conducted using MEGA 11.0 and the Kimura 2-parameter model. A phylogenetic analysis was conducted using Maximum Likelihood (ML) methods on the IQ-TREE web-server (<http://iqtree.cibiv.univie.ac.at/>) using the GTR + G + I substitution model. Tree is drawn and edited using FigTree v.1.4.4 and Adobe Illustrator 2024 (Adobe Inc.).

**4. Results**

**4.1. Taxonomy**

**Family Pseudotyrannochthoniidae Beier, 1932**

**Genus *Spelaeochthonius* Morikawa, 1954**

*Spelaeochthonius* Morikawa 1954: 83–84. Type species: *S. kubotai* Morikawa, 1954

*Allochthonius* (*Spelaeochthonius*): Morikawa 1960: 104–105

*Pseudotyrannochthonius* Muchmore 1967: 132

*Spelaeochthonius* You, Yoo, Harvey and Harms 2022: 135–157

**Diagnosis.** *Spelaeochthonius* is most similar to *Centrochthonius* Beier, 1931 because both genera share the presence of 16 setae on carapace in most species (Fig. 4A, C) whereas other pseudotyrannochthoniid genera (e.g. *Allochthonius*) generally have more than 16 setae (Fig. 4E). *Spelaeochthonius* differs from *Centrochthonius* by having more than six coxal spines on leg coxa I that are also distally elongated (Fig. 4B, D) (You *et al.* 2022). *Spelaeochthonius* also differs from *Allochthonius*, which occurs in the same area, by the number of carapacal setae (16 in *Spelaeochthonius*, 18–28 in *Allochthonius*) (Fig. 4A, E), the form of coxal spines (short and arranged as an oblique line in *Spelaeochthonius*; fan-shaped and on a common tubercle in the genus *Allochthonius*) (Fig. 4B, F), and the size of the intercoxal tubercle (generally larger in *Allochthonius*) (Fig. 4B, F).

**Distribution.** Exclusively found in caves in Japan (six species); China (three species); and South Korea (seven species; but see below).

***Spelaeochthonius dugigulensis* Jeong & Harms sp. nov.**

**Fig. 5–6; Maps 1–2**

**Type material**

Holotype. Male (@@@ - specimen numbers will be added KOREA: Gyeongsangbuk-Province: Uljin-gun, Maehwa-myeon, Dugigul-cave; 36˚53’N 129˚21’E; 25 Sep. 2022; KH Jeong leg.)

Paratypes. three females (@@@ - specimen numbers will be added). Same data as holotype.

**Etymology.** This species is named after the cave “Dugigul” in Uljin­-gun, Gyeongsangbuk-Province, where all specimens were collected.

**Diagnosis.** This species is most similar to *Spelaeochthonius akiyoshiensis* from Japan but both species differ in the following characters: length of chela (1.84mm in *S. dugigulensis* **sp. nov.** and 1.78mm *in S. akiyoshiensis*), number of coxal spines (nine spines vs. 8–9 in *S. akiyoshiensis*), number of setae on cheliceral palm (five vs. six), and the number of marginal teeth in chelal fingers (>30 vs. <30).

**Description**

**Male, adult (holotype, Fig. 5A-B)**

Colour. Uniformly reddish orange.

Chelicera (Fig. 6D). Palm slightly granulate and with five setae, one seta on movable finger; nine marginal teeth on movable finger, eight on fixed finger; two large teeth on the middle of fixed finger; serrula exterior with 25 blades, terminal blades distinctly bigger than basal blades; rallum with 11 pinnate blades; one dorsal lyrifissure.

Pedipalp (Fig. 6E–F). Trochanter 1.83 times, femur 6.69 times, patella 3.49 times, chela 6.04 times, hand 2.26 times longer than broad, movable finger 1.67 times longer than the hand. Fixed finger with eight trichobothria, movable finger with four trichobothria; *isb* and *ib* basally positioned on the dorsum of fixed finger; *eb*, *ist*, and *esb* grouped, *ist* clearly closer to *esb*; *est* and *it* positioned on the middle of fixed finger; *et* positioned next to *xs*, about two areolar apart; *xs* located terminally on the fixed finger; *sb*, *b*, and *t* separately situated from *st*; *st* positioned basally on the movable finger. Fixed finger with 34, movable finger with 36 spaced, apical marginal teeth.

Cephalothorax (Fig. 6A–C). Carapace 0.99 times longer than broad, subquadrate, eyeless; epistome pronounced and triangular; 16 setae on the carapace, three setae pairs on the anterior margin (*Ao*, *Al*, *Am*), setae pair on the lateral margin (*Ol*), three setae pairs on the medial margin (*Om*, *Ml*, *Il*), one setae pair on the posterior margin (*Pm*). Two pairs of anterior lyrifissures, a pair of posterior lyrifissures present. Two acuminate setae on the manducatory process, maxilla with three setae; coxal chaetotaxy 7: 4: 5: 4; coxal spines with nine blades on a low mound; each spine tripartite; bisetose intercoxal tubercle present between coxa III and IV.

Abdomen. Pleural membrane granulate; tergites undivided; tergal chaetotaxy 2: 2: 4: 4: 6: 6: 7: 7: 5: 4: 0: 0; sternite III–V divided; sternal chaetotaxy 12: 21: 12: 10: 10: 9: 10: 8: 5: 2: 2.

Legs. Leg I: trochanter 1.56 times, femur 7.42 times, patella 5.26 times, tibia 5.81 times, tarsus 10.44 times longer than broad. Leg IV: trochanter 1.69 times, femur+patella 4.05 times, tibia 7.73 times, metatarsus 3.76 times, tarsus 10.78 times longer than broad; arolium undivided; pseudotactile seta present on leg IV tarsus and metatarsus.

Male genitalia. 12 setae on the sternite II, six setae on the anterior margin of the genital opening area, eight setae in the genital opening area, 22 setae on the sternite III, and 11 setae on the posterior margin of the genital opening area. Sternite II with two lyrifissures; sternite III with four lyrifissures.

Dimensions (in mm). Body length 2.17. Pedipalp: trochanter 0.39/0.21, femur 1.35/0.20, patella 0.70/0.20, chela 1.84/0.30, hand 0.69/0.30, movable finger 1.15. Chelicera: total 0.64/0.30, hand 0.30/0.30, movable finger 0.34. Leg I: trochanter 0.25/0.16, femur 0.71/0.10, patella 0.45/0.08, tibia 0.37/0.06, tarsus 0.80/0.08. Leg IV: trochanter 0.29/0.17, femur+patella 0.98/0.24, tibia 0.80/0.10, metatarsus 0.32/0.09, tarsus 0.97/0.09.

**Variation (females, paratypes, Fig. 5C–D)**

Same as the holotype.

Pedipalp. Trochanter 1.44–1.79 times, femur 5.05–5.70 times, patella 2.22–2.79 times, chela 5.77–5.80 times, hand 1.91–1.93 times longer than broad, movable finger 2.01–2.02 times longer than the hand. Movable finger with 30–32, fixed finger with 28 marginal teeth.

Cephalothorax. Carapace 0.95–1.10 times longer than broad; with 16 setae; six setae on the anterior margin, and two setae on the posterior margin. Coxal chaetotaxy: 6–7: 4–5: 4: 4–6; coxa I with nine spines.

Abdomen. Tergal chaetotaxy 2: 2: 4: 6: 6: 6–7: 6–7: 7–8: 5–7: 4–5: 0: 0. Sternal chaetotaxy 10–12: 12–14: 12: 10–12: 8–11: 9–12: 8–10: 6–7: 5: 2: 2.

Legs. Leg I: trochanter 1.46–1.52 times, femur 6.58–6.68 times, patella 4.04–4.94 times, tibia 3.74–4.34 times, tarsus 7.11–8.07 times longer than broad; leg IV: trochanter 1.37–1.72 times, femur+patella 3.50–3.97 times, tibia 5.69–6.05 times, metatarsus 2.79–2.94 times, tarsus 6.28–9.50 times longer than broad.

Genitalia. 10–12 setae on the genital opening area; 12–14 setae on sternite III.

Dimension (in mm). Boy length 2.05–2.07. Pedipalp: trochanter 0.30–0.32/0.18–0.21, femur 0.95–1.13/0.19–0.20, patella 0.45–0.59/0.20–0.21, chela 1.55–1.66/0.27–0.29, hand 0.51–0.55/0.27–0.29, movable finger 1.04–1.11. Chelicera: total 0.68–0.72/0.31–0.32, hand 0.37–0.40/0.31–0.32, movable finger 0.38–0.40. Leg I: trochanter 0.21–0.22/0.14–0.15, femur 0.55–0.58/0.08–0.09, patella 0.32–0.38/0.08, tibia 0.24–0.28/0.06–0.07, tarsus 0.42–0.62/0.06–0.08. Leg IV: trochanter 0.25–0.28/0.16–0.18, femur+patella 0.82–0.84/0.21–0.24, tibia 0.64–0.68/0.11, metatarsus 0.21–0.22/0.07–0.08, tarsus 0.40–0.59/0.06.

***Spelaeochthonius geumgulensis* Jeong & Harms sp. nov.**

**Figs 7–8, Maps 1–2**

**Type material**

Holotype. Male (@@@ - specimen numbers will be added KOREA; Chungcheongbuk-Province, Danyang-gun, Danyang-eup, Dodam-ri 47, Geum-cave; 36˚59’N, 128˚51’E; 19 July. 2022; J.H. Oh leg.

Paratype. One female (@@@ - specimen numbers will be added), same data as holotype.

**Etymology.** This species is named after the “Geumgul” cave in Danyang–gun, Chungcheongbuk–Province, where all specimens were collected.

**Diagnosis.** This species is most similar to *Spelaeochthonius dentifer* but differs in the length of femur IV (0.98–1.07mm in *S. geumgulensis* **sp. nov.** vs. 0.97mm in *S. dentifer*), the count of marginal teeth on the fixed chelal fingers (29–33 vs. 34), and ratios of leg IV tarsus (< 10 times longer than broad; > more than 10 times longer than broad).

**Description**

**Male, adult (holotype. Fig. 7A–B)**

Colour. Uniformly pale–orange, legs paler than the body.

Chelicera (Fig. 8D). Cheliceral palm granulate and with six setae, movable finger with one seta in medial position; 15 marginal teeth on the movable finger, seven on the fixed finger; one big tooth on the fixed finger; rallum with 12 blades; serrula exterior with 25 blades, terminal blades bigger than the basal blades; one dorsal lyrifissure.

Pedipalp (Fig. 8E–F). Trochanter 1.73 times, femur 6.15 times, patella 2.77 times, chela (with pedicel) 6.86 times, hand 2.58 times longer than broad, movable finger 1.66 times longer than the hand. Fixed finger with eight trichobothria, movable finger with four trichobothria; *isb* and *ib* situated basally on the dorsum of the fixed finger; *eb*, *ist*, and *esb* grouped, *ist* slightly closer to *esb*; *est* and *it* positioned on the middle of the fixed finger, *it* closely located to *et*; *et* positioned on the middle of *it* and *xs*; *xs* located terminally on the fixed finger; *sb*, *b*, and *t* separately situated from *st*; *st* positioned basally on the movable finger. Both fingers with long and triangular teeth, spaced together; fixed finger with 29 marginal teeth, 30 on the movable finger.

Cephalothorax (Fig. 8A–C). Carapace 1.09 times longer than the broad and subquadrate, posterior margin narrower than the anterior margin; all setae short and acuminate; 16 setae on the carapace, three setae pairs on the anterior margin (*Ao*, *Al*, *Am*), one setae pair on the lateral margin (*Ol*), three setae pairs on the medial margin (*Om*, *Ml*, *Il*), one setae pair on the posterior margin (*Pm*); without eye; epistome small and triangular. Two pairs of anterior lyrifissures, one pair of posterior lyriffisure present. A manducatory process with one acuminate seta; four setae on the maxilla; coxal chaetotaxy 6: 4: 4: 4. Nine spines on the coxa I; each spine with tripartite shape. Intercoxal tubercle distinctly present between coxa III and IV, two setae present.

Abdomen. Pleural membrane granulate. Tergites undivided; tergal chaetotaxy 2: 4: 4: 4: 6: 6: 6: 7: 6: 4: 0: 0. Sternite III, and IV divided; sternal chaetotaxy 13: 22: 16: 12: 12: 10: 10: 7: 6: 2: 2.

Legs. Leg I: trochanter 1.46 times, femur 7.95 times, patella 6.23 times, tibia 4.58 times, tarsus 10.76 times longer than broad. Leg IV: trochanter 1.17 times, femur+patella 4.26 times, tibia 6.22 times, metatarsus 2.66 times, tarsus 8.59 times longer than broad. Arolium undivided.

Male genitalia. 13 setae on the sternite II, six setae situated on the anterior margin of the genital opening area; 22 setae on the sternite III, each five positioned on each side of the opening area. One pair of lyriffisure on sternite II, one pair of lyriffisure on sternite III.

Dimensions (in mm). Body length 2.13. Pedipalp: trochanter 0.37/0.22, femur 1.23/0.20, patella 0.60/0.22, chela (with pedicel) 1.86/0.27, movable finger 1.16, hand 0.70/0.27. Chelicera: total 0.69/0.30, movable finger 0.36, hand 0.63/0.30. Carapace 0.69/0.64. Leg I: trochanter 0.25/0.17, femur 0.74/0.09, patella 0.44/0.07, tibia 0.31/0.07, tarsus 0.74/0.07. Leg IV: trochanter 0.29/0.25, femur+patella 0.98/0.23, tibia 0.74/0.12, metatarsus 0.24/0.09, tarsus 0.57/0.07.

**Variation (female, paratype, Fig. 7C–D)**

Pedipalp. Trochanter 1.70 times, femur 5.66 times, patella 2.92 times, chela (with pedicel) 6.91 times, hand 2.17 times longer than broad, movable finger 1.83 times longer than hand. Movable finger with 32, fixed finger with 31 marginal teeth.

Cephalothorax. Carapace 1.13 times longer than broad, with 16 setae.

Abdomen. Tergal chaetotaxy, 2: 2: 4: 4: 6: 6: 6: 7: 5: 4: 0: 0. Sternal chaetotaxy, 10: 13: 12: 12: 11: 9: 9: 7: 5: 2: 2.

Legs. Leg I: trochanter 1.50 times, femur 7.94 times, patella 5.26 times, tibia 4.35 times, tarsus 13.87 times longer than broad, leg IV: trochanter 1.42 times, femur+patella 4.27 times, tibia 6.15 times, metatarsus 4.33 times, tarsus 7.66 times longer than broad.

Dimensions (in mm). Body length 2.42. Pedipalp: trochanter 0.36/0.21, femur 1.16/0.21, patella 0.61/0.21, chela (with pedicel) 1.88/0.27, movable finger 1.17, hand 0.71/0.27. Chelicera: total 0.70/0.30, movable finger 0.36, hand 0.40/0.30. Carapace 0.74/0.65. Leg I: trochanter 0.24/0.16, femur 0.71/0.09, patella 0.43/0.08, tibia 0.28/0.06, tarsus 0.78/0.06. Leg IV: trochanter 0.27/0.19, femur+patella 1.07/0.25, tibia 0.81/0.13, metatarsus 0.38/0.09, tarsus 0.56/0.07.

***Spelaeochthonius magwihalmigulensis* Jeong & Harms sp. nov.**

**Figs 9–10; Maps 1–2**

**Type material**

Holotype. Female (@@@ - specimen numbers will be added KOREA: Gangwo-Province: Gangneung-si, Okgye-myeon, Nakpung-ri, Magwihalmigul-cave; 37˚37’ N 129˚0’ E; 08 January, 2023; JH Oh leg).

**Etymology.** This species is named after the cave “Magwihalmigul” in the Gangneung-si, Gangwon-Province, where the species was sampled.

**Diagnosis.** This species is most similar to *Spelaeochthonius cheonsooi* by having a small pedipalpal femur (less than 1mm), and a small pedipalpal patella (less than 0.4mm). Both species differ in chelal length (1.49mm *S. cheonsooi* vs. 1.16mm in *S. magwihalmigulensis* **sp. nov.**) and the ratio between the movable chelal finger and the hand (1.67x, in *S. cheonsooi* vs. 2.38x in *S. magwihalmigulensis* **sp. nov.**).

**Description**

**Female, adult (holotype, Fig. 9A–B)**

Colour. Body pale-orange; chelicera darker than the body.

Chelicera (Fig. 10D). Coarsely granulate; five setae on the cheliceral palm, one seta on the movable finger; nine marginal teeth on the movable finger, six on the fixed finger; one big tooth on the fixed finger; serrula exterior with 16 blades; rallum with 11 blades; one dorsal lyrifissure

Pedipalp (Fig. 10E–F). Trochanter 1.59 times, femur 5.51 times, patella 1.43 times, chela 6.78 times, hand 2 times longer than broad, movable finger 2.38 times longer than the hand. Fixed finger with eight trichobothria, movable finger with four trichobothria; *isb* and *ib* basally positioned on the dorsum of the fixed finger; *eb*, *ist*, and *esb* grouped, *ist* positioned middle of *eb* and *esb*; *est* and *it* positioned on the middle of the fixed finger; *et* positioned between *xs* and *it*, but slightly closer the *xs*; *xs* located terminally on the fixed finger; *sb*, *b*, and *t* grouped, each apart about one areolar; *sb*, *b*, and *t* separately situated from *st*; *st* positioned basally on the movable finger. Fixed finger with 25, movable finger with 27 marginal teeth, spaced together; apical shape in anterior teeth, retrose and rounded teeth basally positioned on movable finger.

Cephalothorax (Fig. 10A–C). Carapace 0.90 times longer than the broad; lateral margin distinctly wider than the base; eyeless; epistome big and triangular; 16 setae on the carapace, three setae pairs on the anterior margin (*Ao*, *Al*, *Am*), setae pair on the lateral margin (*Ol*), three setae pairs on the medial margin (*Om*, *Ml*, *Il*), one setae pair on the posterior margin (*Pm*); seta short and acuminate. Two pairs of anterior lyrifissures, a pair of medial lyrifissures, a pair of posterior lyrifissures present. One acuminate seta on the manducatory process, three setae on the maxilla; coxal chaetotaxy 5: 4: 3: 3; coxal spines with eight blades, each blade tripartite; intercoxal tubercle distinct and bisetose.

Abdomen. Pleural membrane granulate; tergites undivided; tergal chaetotaxy 2: 2: 4: 4: 6: 6: 8: 8: 6: 4: 0: 0; sternites IV–V divided; sternal chaetotaxy 9: 10: 10: 10: 9: 10: 6: 6: 4: 2: 2.

Legs. Leg I: trochanter 1.56 times, femur 5.47 times, patella 1.43 times, tibia 4.6 times, tarsus 10.2 times longer than broad; leg IV: trochanter 1.78 times, femur+patella 5.13 times, tibia 4.19 times, metatarsus 3.33 times, tarsus 9.90 times longer than the hand. Arolium undivided. Pseudotactile seta situated basally on leg IV tarsus and metatarsus.

Female genitalia. Nine setae on the sternite II, ten setae on the sternite III.

Dimensions (in mm). Body length 1.26. Pedipalp: trochanter 0.24/0.15, femur 0.91/0.17, patella 0.33/0.23, chela 1.16/0.17, hand 0.34/0.17, movable finger 0.82. Chelicera: total 0.64/0.28, hand 0.32/0.28, movable finger 0.32. Leg I: trochanter 0.18/0.11, femur 0.48/0.09, patella 0.27/0.07, tibia 0.23/0.05, tarsus 0.51/0.05. Leg IV: trochanter 0.27/0.15, femur+patella 0.72/0.14, tibia 0.23/0.05, metatarsus 0.24/0.07, tarsus 0.52/0.05.

***Spelaeochthonius yamigulensis* Jeong & Harms sp. nov.**

**Figs 11–12; Map 1–2**

**Type material**

Holotype. Male (@@@ - specimen numbers will be added KOREA: Gangwon-Province: Jeongseon-gun, Najeon-ri, San 183-2, Yamigul-cave; 37˚25’ N 128˚38’ E; 13 Nov. 2022; K.-H. Jeong leg.)

**Etymology.** This species is named after the “Yamigul” cave in Jeongseon-gun, Gangwon-Province, where the species was collected.

**Diagnosis.** The species differs from other congeners by the number of carapacal setae. In contrast to other species within the genus, where seta *pl* is absent, in *S. yamigulensis* **sp. nov.** seta *pl* is present and there are 18 carapacal setae instead of 16 setae which is the standard for *Spelaeochthonius*.

**Description.**

**Male, adult (holotype, Fig. 11A–B)**
Colour. Body brown; chela pale-orange, darker at the tip.

Chelicera (Fig. 12D). Cheliceral palm smooth; five setae on cheliceral palm, one on the movable finger; nine marginal teeth on the fixed finger, movable finger with nine marginal teeth; three big teeth on the terminal of the fixed finger; rallum with blades; serrula exterior with blades; one dorsal lyrifissure.

Pedipalp (Fig. 12E–F). Trochanter 1.65 times, femur 7.04 times, patella 2.45 times, chela 5.46 times, hand 2.19 times longer than broad, movable finger 1.49 times longer than the hand. Fixed finger with eight trichobothria, movable finger with four trichobothria; *isb* and *ib* basally positioned on the dorsum of fixed finger; *eb*, *ist*, and *esb* grouped, *ist* positioned slightly closer to *esb*; *est* and *it* positioned on the middle of fixed finger; *et* positioned near *xs*, about three areolar apart; *xs* located terminally on the fixed finger; *sb*, *b*, and *t* separately situated from *st*; *st* positioned basally on the movable finger. Fixed finger with 15, movable finger with 18 marginal teeth; apical teeth in both fingers.

Cephalothorax (Fig. 12A–C). Carapace 1.02 times longer than broad; anterior margin slightly wider than posterior margin; 18 setae on the carapace, three setae pairs on the anterior margin (*Ao*, *Al*, *Am*), one setae pair on the lateral margin (*Ol*), three setae pairs on the medial margin (*Om*, *Ml*, *Il*), two setae pairs on the posterior margin (*Pl, Pm*); seta acuminate. A pair of anterior lyrifissures, a pair of medial lyrifissures, two pairs of posterior lyrifissures present. Two acuminate setae on the manducatory process, three setae on the maxilla; coxal chaetotaxy 7: 4: 4: 4; coxal spines with eight blades; each blade tripartite; intercoxal tubercle distinct, two setae on the tubercle.

Abdomen. Pleural membrane granulate; tergites undivided; tergal chaetotaxy 2: 4: 4: 4: 5: 6: 6: 7: 7: 5: 4: 0: 0; sternite III divided, sternite IV partially divided; sternal chaetotaxy 9: 28: 14: 12: 15: 13: 11: 11: 8: 6: 2: 2.

Legs. Leg I: trochanter 1.48 times, femur 5.43 times, patella 5.17 times, tibia 3.93 times, tarsus 10.1 times longer than broad; leg IV: trochanter 1.33 times, femur+patella 4.86 times, tibia 6.31 times, tarsus 3.51 times, metatarsus 12.31 times longer than broad. Arolium undivided. Pseudotactile seta present on leg IV tarsus.

Male genitalia. Sternite II with nine setae; sternite III with 28 setae, 15 setae near the genital opening area. Sternite II with two lyrifissures; sternite III with six lyrifissures.

Dimensions (in mm). Body length 1.72. Pedipalp: trochanter 0.28/0.17, femur 1.04/0.15, patella 0.43/0.18, chela 1.56/0.29, hand 0.63/0.29, movable finger 0.93. Chelicera: total 0.59/0.23, hand 0.30/0.23, movable finger 0.29. Leg I: trochanter 0.22/0.15, femur 0.46/0.08, patella 0.38/0.07, tibia 0.28/0.07, tarsus 0.65/0.06. Leg IV: trochanter 0.20/0.15, femur+patella 0.80/0.17, tibia 0.59/0.09, metatarsus 0.26/0.07, tarsus 0.73/0.06.

**Remarks.** This species has a carapacal setation that differs from the diagnosis for *Spelaeochthonius* presented in You et al. (2022). The genus generally has 16 setae that are arranged s4s: 4: 2: 2: 2: 2 (Hou and Zhang 2024) but in *S. yamingulensis* sp. nov. seta *pl* is present and the pattern is 4: 2: 2: 2: 4. Similar derivations are known in other pseudotyrannochthoniid genera and an interesting analogy is *Centrochtonius anatonus* Harvey & Harms, 2022 that shows the same duplication and has 18 carapacal setae (*pl* present) whereas all other species have 16 setae (*pl* absent) (Harms & Harvey, 2022). Genetically but also in terms of coxal spine morphology, *S. yamingulensis* **sp. nov.** can clearly be placed in *Spelaeochthonius* and the number of posterior carapaceal setae seems to be somewhat variable within both genera.

**4.2. Molecular analyses**

The Maximum Likelihood (ML) phylogenetic analysis of the mtCOI dataset supported the species hypothesis that was derived from morphology and suggested the presence of at least seven species of *Spelaeochthonius* in South Korea (Fig. 12). Although the bootstrap values were generally low at the basal nodes it appears that the Korean fauna of *Spelaeochthonius* is polyphyletic and that two species from Japan nested within the Korean fauna. Overall, the terminals can be classified into three genetic clades (A–C) that differ in terms of pairwise genetic distances that range between 13.7% to 19.8%. In clade B, the pairwise genetic distance between *S. cheonsooi* and *S. yamigulensis* **sp. nov.** is 10.8% and the pairwise genetic distance between *S. kobayashii* and *Spelaeochthonius* sp. equals 10%. In clade C, the genetic distance between *S. dentifer* and *S. magwihalmigulensis* **sp. nov.** is 6.8% and in *S. seungsookae* and *S. geumgulensis* **sp. nov.** is 15.6–16% (Table. 1).

According to Cosgrove et al. (2016), >10% COI divergences might be used for identifying putative species in Pseudoscorpiones at a molecular level. However, the COI divergences between *S. dentifer* and *S. magwihalmigulensis* **sp. nov.** are lower (6.8%) but both species are clearly differentiated morphologically and differ in the number of teeth on both the fixed and movable chelal finger (25/27 in *S. magwihalmigulensis* **sp. nov.** vs 34/37 in *S. dentifer*), the ratio between the movable chelal finger and the hand (1.67 in *S. magwihalmigulensis* **sp. nov.** vs 2.17 in *S. dentifer*), and ratios of the chelal length (1.16mm in *S. magwihalmigulensis* **sp. nov.** vs 1.60 mm in *S. dentifer*). Both species also originate from different karst systems that are approximately 47 km apart from each other and not interconnected.

Table 1. Genetic divergences within all species included in this study

| 1) Intraspecific genetic divergence | | | | | | | | | |
| --- | --- | --- | --- | --- | --- | --- | --- | --- | --- |
| Species  (Comparison pairs) | | | | Intraspecific divergence | | | | | |
|  |  |  |  | Maximum | | Minimum | | Average | |
| ***S. cheonsooi*** | | | | – | | – | | – | |
| ***S. dentifer*** | | | | – | | – | | – | |
| ***S. geumgulensis* sp. n.** (n=2) | | | | 0.6% | | 0.6% | | 0.6% | |
| ***S. dugigulensis* sp. n.** (n=4) | | | | 0.17% | | 0 | | 0.08% | |
| ***S. magwihalmigulensis* sp. n.** | | | | – | | – | | – | |
| ***S. seungsookae*** | | | | – | | – | | – | |
| ***S. yamigulensis* sp. n.** | | | | – | | – | | – | |
| 2) Interspecific genetic divergence | | | | | | | | | |
| Species | ***S. dentifer*** | ***S. kobayashii*** | ***S. seungsookae*** | ***S. geumgulensis* sp.n** | ***S. dugigulensis* sp.n.** | | ***S. magwihalmigulensis* sp.n.** | | ***S. yamigulensis* sp.n.** |
| ***Spelaeochthonius* sp.** | 18.8% | 10.0% | 17.2% | 19.1–19.8% | 14.7–14.9% | | 15.9% | | 16.6% |
| ***S. cheonsooi*** | 18.1% | 16.4% | 18.7% | 18.8–19.2% | 16.2–16.4% | | 14.9% | | 10.8% |
| ***S. dentifer*** |  | 16.3% | 14.7% | 18.9–19.3% | 16.3–16.4% | | 6.8% | | 17.4% |
| **.*S. kobayashii*** |  |  | 15.5% | 18.5–19.1% | 15.5% | | 13.5% | | 16.2% |
| **.*S. seungsookae*** |  |  |  | 15.6–16.0% | 15% | | 12.7% | | 17.2% |
| ***S. geumgulensis* sp.n.** |  |  |  |  | 16.0–16.9% | | 17.6–18.1% | | 19.2–19.8% |
| ***S. dugigulensis* sp.n.** |  |  |  |  |  | | 13.7–13.9% | | 15.9–16.0% |
| ***S. magwihalmigulensis* sp.n.** |  |  |  |  |  | |  | | 14.7% |

**5. Discussion**

**5.1. Species diversity of *Spelaeochthonius* in South Korea**

*Spelaeochthonius* is a genus found exclusively in caves habitat of eastern Asia and the 15 described species in this genus are altogether blind, pale, and strongly troglobitic with the exception of the recently discovered *S. tuoliangensis* from China that retains residual eyes and shorter legs (Hou & Zhang, 2024). Collecting pseudoscorpions in caves is usually by chance and until recently, very few surveys have specifically targeted troglobite pseudoscorpions in eastern Asia. The fauna of South Korea is no exception and two target surveys with limited spatial sampling (You et al. 2022; this study) have not only resulted in the discovery of new species, but also revealed that almost every cave system visited by the first author had a cave-adapted pseudoscorpion fauna that was completely unknown before. The Korean Peninsula has karstic formations sculptured on most major mountain ranges, such as Taebaek and Sobaek Mountain ranges, primarily in the eastern regions, and karstic studies indicate that there are more than 1,000 caves in South Korea alone (Woo & Kim 2018). The present study examined four cave populations, which exhibited distinct morphological and genetic differences from other cave populations. Based on these results, it is not difficult to perceive a hypothesis by which the pseudoscorpion fauna of Korean caves will be highly diverse and comprise potentially dozens of new species (Lee & Min 2021; Oh et al. 2023). Like similar cases in neighboring countries such as China, the pseudoscorpion biodiversity of Korean subterranean ecosystem is significantly higher than previously thought, underscoring the necessity for further research into these systems (Feng et al. 2020; Hou et al. 2022; Gao et al. 2023). To summarize, discovering undescribed subterranean species is an important project for conserving and understanding the unveiled fauna of this dark ecosystem.

**5.2. Endemism and biogeography**

Due to the limited number of specimens that are usually collected during field work in caves, it is often challenging to infer intraspecific genetic divergences within cave species and total species ranges. The pseudoscorpion species in our present study are no different but the results demonstrate a clear distinction between the intraspecific and interspecific distances, which is consistent with the concept of the ‘barcode gap’ in molecular taxonomy suggesting that the variation found across species is higher than the genetic variability seen within a species (Puillandre et al. 2011). Interspecific genetic distances in our study ranged from 6.8% to 19.8% but intraspecific distances observed in our study were always smaller than 0.6%. These results could be caused by under-sampling (e.g. only one cave in a complex karst system was sampled) but might also be real considering that genetic drift in cave populations often results in a reduction or loss of genetic variability (Avise & Selander 1972; Carlini et al. 2009; Bradic et al. 2012; Wang et al. 2017; Trontelj 2018). It has also been observed frequently that gene flow between caves is often scarce and often results in the formation of numerous cryptic or closely related species (Caccone 1985; Rastorgueff et al. 2014; Trontelj 2018; Balogh et al. 2020; Oh et al. 2022). The same has been recorded in non-phoretic pseudoscorpions (e.g. Harrison et al. 2014; Hlebec et al. 2023) and our data are in line with such studies and suggest that scarce gene flow and genetic drift in cave populations could foster endemism in cave pseudoscorpions across the Korean Peninsula. This hypothesis could be explored further using multi-gene phylogenetic studies involving nuclear molecular markers, or even genomics, and detailed specimen collections in multiple caves belonging to a single karst system. In the context of genetic data we reiterate once more that the COI pairwise divergences between *S. dentifer* and *S. maguihalgulensis* **sp. nov.** are somewhat lower than in previous studies (Cosgrove et al. 2016). However, morphological differences and distributional patterns support the hypothesis that they represent different species and other (nuclear) genetic markers might also recover these divergences. Also, the previous research was conducted with epigean species, so that the genetic distance could be different between the two examples.

Although only based on single-gene analyses, our study also suggests close affinities of the Southern Korean fauna of *Spelaeochthonius* to Japan; a result that was also recovered during a global phylogenetic study of Pseudotyrannochthoniidae using multiple genetic markers (Harms et al. 2024). Two hypotheses can be evoked to explain the faunal affinities between the Korean Peninsula and the Japanese Archipelago. First, this genus might have been widely distributed throughout East Asia before the formation of the Sea of Japan. Alternatively, there might have been faunal exchange during the last glacial maximum when sea levels were lower and land connections existed between Japan and Korea (Kimura 1996; Lee et al. 2014). More extensive field sampling and comprehensive phylogenetic analyses are necessary to test these hypotheses rigorously but the data presented in Harms et al. (2024) suggest that these relationships are old and certainly predating the last glacial periods.

**5.3. Caves as a major contributor to Korean diversity and conservation values**

Caves in Korea support diverse communities of invertebrates, including pseudoscorpions. According to Kim et al. (2004), at least 260 species of invertebrates that belong to four phyla, 31 orders and 94 families are known from caves in Korea. There are 97 species of spiders alone and the known cave diversity represents 14% of all spider species found in South Korea (Kim 2005) which is a substantial number considering the poor state of cave research in Korea. Of the 33 pseudoscorpion species now recorded in Korea, eight species (24%) are troglobites and this number will surely rise in the future.

Cave pollution and the destruction of caves are unfortunately common problems in South Korea, with sometimes devastating consequences. For example, the carabid beetle *Coreoblemus parvicollis* Uéno, 1969 was known only from Cheongpungpunghyeol Cave and last sighted in 1967 before the completion of the Chungju-dam. The species is now listed as extinct (IUCN 2024). According to Kim & Kim (2007), several larva caves on Jeju Island are under severe destruction thread because of road construction and pollution by tourists. It is clear that the emerging karst biodiversity in Korea is underrepresented in practical nature conservation and presently underappreciated and not at all protected by conservation legislation. Target surveys in combination with taxonomy and distribution modelling for cave fauna in South Korea are suggested to improve both knowledge and practical conservation of cave fauna across the country.

A small number of specimens were included in this study (four specimens for *S. dugigulensis* **sp. nov.**, two specimens for *S. geumgulensis* **sp. nov.**, and one specimen each for *S. magwihalmigulensis* **sp. nov.** and *S. yamigulensis* **sp. nov.**). Because of the rarity of subterranean species and the danger of accessing caves, it is common to use a limited number of specimens for descriptions (Gao et al., 2018, 2023). And, considering the cryptic diversity of subterranean pseudoscorpion fauna in South Korea, the description of unknown species is an urgent task. Taxonomic knowledge and biological background are critical for conservation efforts, helping to prevent 'dark extinction' of these cryptic animals (Taylor et al., 2018; Thomson et al., 2018; Woinarski et al., 2024).

**6. Acknowledgements**

KHJ would like to thank Yong-Gun Choi (The Korean Institute of Biospeleology) for guiding caves, and Dongyoung Kim and Jonghwa Oh (Seoul National University) for assistance in the field.

**7. References**

1. Avise JC, Selander RK. Evolutionary genetics of cave-dwelling fishes of the genus *Astyanax*. Evolution. 1972; 26: 1–19. doi: <https://doi.org/10.1111/j.1558-5646.1972.tb00170.x>
2. Balogh A, Ngo L, Zigler KS, Dixon G. Population genomics in two cave-obligate invertebrates confirms extremely limited dispersal between caves. Scientific Reports. 2020; 10(1): 17554. doi: <https://doi.org/10.1038/s41598-020-74508-9>
3. Bradic M, Beerli P, García-de León FJ, Esquivel-Bobadilla S, Borowsky RL. Gene flow and population structure in the Mexican blind cavefish complex (*Astyanax mexicanus*). *BMC* Evolutionary Biology. 2012; 12: 1–17. doi: <https://doi.org/10.1186/1471-2148-12-9>
4. Carlini DB, Manning J, Sullivan PG, Fong DW. Molecular genetic variation and population structure in morphologically differentiated cave and surface populations of the freshwater amphipod *Gammarus minus*. Molecular Ecology. 2009; 18(9): 1932–1945. doi: <https://doi.org/10.1111/j.1365-294X.2009.04161.x>
5. Chamberlin JC. The arachnid order Chelonethida. Stanford University Publications, Biological Sciences. (1931); 7: 1–284.
6. Chang GD, Son MW, Beak SA, Lee TG, Keum E, Choi Y, Park KH. Correlation between environmental factors and springtail species (Hexapoda: Collembola) inhabiting Jangamgul Cave of Korea. Journal of Asia-Pacific Biodiversity. 2022; 15(3): 336–344. doi: <https://doi.org/10.1016/j.japb.2022.04.012>
7. Cosgrove JG, Agnarsson I, Harvey MS, Binford GJ. Pseudoscorpion diversity and distribution in the West Indies: sequence data confirm single island endemism for some clades, but not others. Journal of Arachnology. 2016; 44(3): 257–271. doi: <https://www.jstor.org/stable/24892348>
8. Feng Z, Wynne JJ, Zhang F. Cave-dwelling pseudoscorpions of China with descriptions of four new hypogean species of *Parobisium* (Pseudoscorpiones, Neobisiidae) from Guizhou Province. Subterranean Biology. 2020; 34: 61–98. doi: <https://doi.org/10.3897/subtbiol.34.49586>
9. Gao Z, Wynne JJ, Zhang F. Two new species of cave-adapted pseudoscorpions (Pseudoscorpiones: Neobisiidae, Chthoniidae) from Guangxi, China. The Journal of Arachnology. 2018; 46(2): 345–354. doi: <https://doi.org/10.1636/JoA-S-17-063.1>
10. Gao Z, Hou Y, Zhang F. Four new species of cave-adapted pseudoscorpions (Pseudoscorpiones, Pseudotyrannochthoniidae) from Guizhou, China. ZooKeys. 2023; 1139: 33–69. doi: <https://www.doi.org/10.3897/zookeys.1139.96639>
11. Harms D, Harvey MS, Roberts JD, Loria SF. Tectonically driven climate change and the spread of temperate biomes: Insights from dragon pseudoscorpions (Pseudotyrannochthoniidae), a globally distributed arachnid lineage. Journal of Biogeography. 2024; 51: 1032–1048. doi: <https://doi.org/10.1111/jbi.14801>
12. Harrison SE, Guzik MT, Harvey MS, Austin AD. Molecular phylogenetic analysis of Western Australian troglobitic chthoniid pseudoscorpions (Pseudoscorpiones: Chthoniidae) points to multiple independent subterranean clades. Invertebrate Systematics. 2014; 28(4): 386–400 doi: <https://doi.org/10.1071/IS14005>
13. Harvey MS. Catalogue of the Pseudoscorpionida. Manchester University Press, Manchester. 1991.
14. Hlebec D, Harms D, Kučinić M, Harvey MS. Integrative taxonomy of the pseudoscorpion family Chernetidae (Pseudoscorpiones: Cheliferoidea): evidence for new range-restricted species in the Dinaric Karst. Zoological Journal of the Linnean Society. 2023; 200(3): 644–669. doi: <https://doi.org/10.1093/zoolinnean/zlad083>
15. Hlebec D, Podnar M, Kučinić M, Harms D. Molecular analyses of pseudoscorpions in a subterranean biodiversity hotspot reveal cryptic diversity and microendemism. Scientific Reports. 2024; 13(1): 430. doi: <https://doi.org/10.1038/s41598-022-26298-5>
16. Hou Y, Gao Z, Zhang F. Two new species of cave-adapted pseudoscorpions (Pseudoscorpiones, Chthoniidae) from Yunnan, China. ZooKeys. 2022; 1097: 65–83. doi: <https://doi.org/10.3897/zookeys.1097.82527>
17. Hou Y, Zhang F. Three new species of dragon pseudoscorpions (Pseudoscorpiones, Pseudotyrannochthoniidae) from China. ZooKeys. 2024; 1204: 135–154. doi: <https://doi.org/10.3897/zookeys.1204.111842>
18. IUCN. The IUCN Red List of Threatened Species. Version 2024-2. 2024. <https://www.iucnredlist.org>. Accessed on [January 22, 2025].
19. Judson ML. A new and endangered species of the pseudoscorpion genus *Lagynochthonius* from a cave in Vietnam, with notes on chelal morphology and the composition of the Tyrannochthoniini (Arachnida, Chelonethi, Chthoniidae). Zootaxa. 2007; 1627: 53–68. doi: <https://doi.org/10.11646/zootaxa.1627.1.4>
20. Jeong KH, Harms D, & Yoo JS. New species of the pseudoscorpion genus *Pararoncus* (Pseudoscorpiones, Syarinidae) from Korea. Evolutionary Systematics. 2024a; 8: 91–100. doi: <https://doi.org./10.3897/evolsyst.8.123108>
21. Jeong KH, Harms D, Yoo JS. The pseudoscorpion genus Nipponogarypus (Pseudoscorpiones, Olpiidae) found in seashore habitats in Japan and Korea. Zoosystematics and Evolution. 2024b; 100(3): 1053–1060. doi: <https://doi.org./10.3897/zse.100.123213>
22. Kim D, Hirt MV, Won YJ, Simons AM. Small fishes crossed a large mountain range: Quaternary stream capture events and freshwater fishes on both sides of the Taebaek Mountains. Integrative Zoology. 2017; 12(4): 292–302. doi: <https://doi.org/10.1111/1749-4877.12228>
23. Kim BW, Choi YG, Soh HY, Lee H, Kim WR, Lee WC. A list of cave fauna and research prospect in Korea. Korean Journal of Environmental Biology. 2004; 22(1): 12–27.
24. Kim JP. Check list of Korean Araneae spiders. Korean Arachnology. 2005; 21: 75–154.
25. Kim SI, Park SK, Lee H, Oshida T, Kimura J, Kim YJ, Nguyen ST, Sashika M, Min MS. Phylogeography of Korean raccoon dogs: implications of peripheral isolation of a forest mammal in East Asia. Journal of Zoology. 2013;290(3): 225–235. doi: <https://doi.org/10.1111/jzo.12031>
26. Kim B, Kim T. Conservation and Management of Lava Tubes in Jeju Island, Korea. Journal of the Korean Association of Regional Geographers*.* 2007; 13(6): 609–622.
27. Kimura M. Quaternary paleogeography of the Ryukyu Arc. Journal of Geography (Chigaku Zasshi). 1996*;* 105(3): 259–285. doi: <https://doi.org/10.5026/jgeography.105.3_259>
28. Kolesnikov VB, Mikhailov KG, Turbanov IS. A new species of the genus *Centrochthonius* Beier, 1931 (Pseudoscorpiones: Pseudotyrannochthoniidae) from Kyrgyzstan. Arthropoda Selecta. 2023; 32(4): 444–454. doi: <https://doi.org/10.15298/arthsel.32.4.08>
29. Lee JH, Lee DH, Choi IS, Choi BH. Genetic diversity and historical migration patterns of an endemic evergreen oak, *Quercus acuta*, across Korea and Japan, inferred from nuclear microsatellites. Plant Systematics and Evolution. 2014; 300: 1913–1923. doi: <https://doi.org/10.1007/s00606-014-1017-9>
30. Lee CW, Min GS. Three new species of subterranean amphipods (Pseudocrangonyctidae: *Pseudocrangonyx*) from limestone caves in South Korea. PeerJ. 2021; 9: e10786. doi: <https://doi.org/10.7717/peerj.10786>
31. Min MS, Yang SY, Bonett RM, Vieites DR, Brandon RA, Wake DB. Discovery of the first Asian plethodontid salamander. Nature. 2005; 435(7038): 87–90. doi: <https://doi.org/10.1038/nature03474>
32. Morikawa K. Results of the speleological survey in South Korea 1966. XX. New pseudoscorpions from South Korea. Bulletin of the National Science Museum of Tokyo. 1970; 13: 141–148.
33. Muster C, Spelda J, Rulik B, Thormann J, von der Mark L, Astrin JJ. The dark side of pseudoscorpion diversity: The German Barcode of Life campaign reveals high levels of undocumented diversity in European false scorpions. Ecology and Evolution. 2021; 11(20): 13815–13829. doi: <https://doi.org/10.1002/ece3.8088>
34. Oh JH, Kim S, Lee S. DNA barcodes reveal population-dependent cryptic diversity and various cases of sympatry of Korean leptonetid spiders (Araneae: Leptonetidae). Scientific Reports. 2022; 12(1): 15528. doi: <https://doi.org/10.1038/s41598-022-18666-y>
35. Oh JH, Choi YG, Lee S. The first record of Telemidae (Arachnida, Araneae) from Korea, with a description of new species of the genus Telema Simon, 1882. Journal of Asia-Pacific Entomology. 2023; 26(2): 102088. doi: <https://doi.org/10.1016/j.aspen.2023.102088>
36. Ohira H, Sato K, Tsutsumi T, Kaneko S, Choi HJ. DNA barcoding suggested the existence of cryptic species and high biodiversity of South Korean pseudoscorpions (Arachnida, Pseudoscorpiones). Journal of Asia-Pacific Biodiversity. 2018; 11(3): 399–407. doi: <https://doi.org/10.1016/j.japb.2018.04.005>
37. Puillandre N, Lambert A, Brouillet S, Achaz G. ABGD, Automatic Barcode Gap Discovery for primary species delimitation. Molecular Ecology. 2012; 21(8): 1864–1877. doi: <https://doi.org/10.1111/j.1365-294X.2011.05239.x>
38. Tamura K, Stecher G, and Kumar S. MEGA11: Molecular Evolutionary Genetics Analysis version 11. Molecular Biology and Evolution. 2021; 38: 3022–3027. doi: <https://doi.org/10.1093/molbev/msab120>
39. Taylor GS, Braby MF, Moir ML, Harvey MS, Sands DP, New TR, et al. Strategic national approach for improving the conservation management of insects and allied invertebrates in Australia. Austral Entomology. 2018; 57(2): 124-149. doi: <https://doi.org/10.1111/aen.12343>
40. Trifinopoulos J, Nguyen LT, von Haeseler A, Minh BQ. W-IQ-TREE: a fast online phylogenetic tool for maximum likelihood analysis. Nucleic acids research. 2016; 44(W1): W232-W235. doi: <https://doi.org/10.1093/nar/gkw256>
41. Trontelj P. Structure and genetics of cave populations. Cave Ecology*. 2018;* 235: 269–295. doi: <https://doi.org/10.1007/978-3-319-98852-8_12>
42. Wang J, Zhang H, Xie F, Wei G, Jiang JP. Genetic bottlenecks of the wild Chinese giant salamander in karst caves. Asian Herpetological Research. 2017; 8(3): 174–18. doi: 10.16373/j.cnki.ahr.170038
43. Woo KS, Kim L. Geoheritage evaluation of caves in Korea: A case study of limestone caves. Geoheritage. 2018: 373¬386. doi: <https://doi.org/10.1016/B978-0-12-809531-7.00021-6>
44. World Pseudoscorpiones Catalog. World Pseudoscorpiones Catalog, Natural History Museum Bern. 2022. online at <http://wac.nmbe.ch>, accessed on [January 15, 2025].
45. Woinarski JC, Braby MF, Gibb H, Harvey MS, Legge SM, Marsh JR., et al. This is the way the world ends; not with a bang but a whimper: Estimating the number and ongoing rate of extinctions of Australian non-marine invertebrates. Cambridge Prisms: Extinction. 2024; 2: e23. doi: <https://doi/org/10.1017/ext.2024.26>
46. You J, Yoo JS, Harvey MS, Harms D. Some cryptic Korean karst creatures: revalidation of the pseudoscorpion genus *Spelaeochthonius* (Pseudoscorpiones: Pseudotyrannochthoniidae) and description of two new species from Korea. *Journal of Arachnology*. 2022; 50(2): 135–157. doi:<https://doi.org/10.1636/JoA-S-21-025>
