# Supplementary material for: Four new species of dragon pseudoscorpions (Pseudoscorpiones: Pseudotyrannochthoniidae: Spelaeochthonius) from caves in South Korea revealed by integrative taxonomy
Source: PLoS One. 2025 Jul 9;20(7):e0325375. doi: 10.1371/journal.pone.0325375 (PMC12240370; doi:10.1371/journal.pone.0325375)
Supplement: Attachment — Submitted filename: Response to Reviewers.docx [file pone.0325375.s004.docx]

Manuscript Number: PONE-D-25-09020

**Dear Editors**

Thank you for giving us the opportunity to resubmit the revised manuscript to PLOS One. We feel thankful to you, and the reviewer providing valuable feedback on the manuscript. We have incorporated the necessary changes in response to the reviewers’s comment.

Here is the point-by-point response to the reviewer’s comments and suggestions.

**Reviewer #1: Dr. Jithin Johnson**

Thank you for providing valuable advise on the manuscript. We changed every typos you pointed out, and here are some comments and responses.

**Comments #1,** typos

**Response:** We changed every typos that reviewer pointed out. Thank you for your sharped suggestion.

**Comments #2,** problems in the “References”

**Response:** Thank you for your suggestion. We revised everything you mentioned.

**Reviewer #2: Dr. Feng Zhang**

Thank you for providing valuable feedback on the manuscript. We changed and revised every typos you suggested, and here are some comments and responses.

**Comments #1,** problems in the “References”

**Response:** We added every absent references. Also, we deleted the reference “Lee et al., 2008”.

**Comments #2,** problems in the “*Spelaeochthonius dugigulensis*”

**Response:** Thank you for your detailed revision. Firstly, we recalculated the ratios of appendages and revised them accurately. Also, we checked the differences between *S. dugigulensis* sp. nov. and *S. akiyoshiensis* and changed the length to L/W ratios of chela, and deleted the number of coxal spines.

**Comments #3,** problems in the “Figure 1”

Response: We added distributions of the missing species, “*Spelaeochthonius tuoliangensis* and *S. huanglaoensis*”.
